# Vasoactive Intestinal Peptide modulates trophoblast-derived cell line function and interaction with phagocytic cells through autocrine pathways

**DOI:** 10.1038/srep26364

**Published:** 2016-05-23

**Authors:** Daiana Vota, Daniel Paparini, Vanesa Hauk, Ayelén Toro, Fatima Merech, Cecilia Varone, Rosanna Ramhorst, Claudia Pérez Leirós

**Affiliations:** 1 Laboratory of Immunopharmacology, Department of Biological Chemistry, School of Sciences, University of Buenos Aires, IQUIBICEN-CONICET, Ciudad Universitaria, Pab. 2, (1428) Buenos Aires, Argentina; 2 Laboratory of Placental Molecular Physiology, Department of Biological Chemistry, School of Sciences, University of Buenos Aires, IQUIBICEN-CONICET, Ciudad Universitaria, Pab. 2, (1428) Buenos Aires, Argentina

## Abstract

Trophoblast cells migrate and invade the decidual stroma in a tightly regulated process to maintain immune homeostasis at the maternal-placental interface during the first weeks of pregnancy. Locally synthesized factors modulate trophoblast cell function and their interaction with maternal leukocytes to promote the silent clearance of apoptotic cells. The vasoactive intestinal peptide (VIP) is a pleiotropic polypeptide with trophic and anti-inflammatory effects in murine pregnancy models. We explored the effect of VIP on two human first trimester trophoblast cell lines, particularly on their migration, invasiveness and interaction with phagocytic cells, and the signalling and regulatory pathways involved. We found that VIP enhanced trophoblast cell migration and invasion through the activation of high affinity VPAC receptors and PKA-CRE signalling pathways. VIP knocked-down trophoblast cells showed reduced migration in basal and leukemic inhibitor factor (LIF)-elicited conditions. In parallel, VIP-silenced trophoblast cells failed to induce the phagocytosis of apoptotic bodies and the expression of immunosuppressant markers by human monocytes. Our results suggest that VIP-mediated autocrine pathways regulate trophoblast cell function and contribute to immune homeostasis maintenance at placentation and may provide new clues for therapeutic intervention in pregnancies complicated by defective deep placentation.

Trophoblast cells migrate and invade the decidual stroma in a tightly regulated process to maintain immune homeostasis during the first weeks of pregnancy^1^,^2^. Migration, invasion and trophoblast interaction with nearby cells is modulated by local maternal and placental factors to achieve deep placentation with almost complete transformation of spiral arteries. The overall process highly depends on trophoblast cell differentiation and their appropriate communication with maternal leukocytes which are recruited in large amounts to the maternal-placental interface^3^. A defective invasion capacity of trophoblast cells with absent or incomplete vascular remodelling and an excessive apoptosis of trophoblast cells that are not efficiently removed by phagocytosis characterize life threatening pregnancy complications such as preeclampsia (PE) and intrauterine growth restriction (IUGR)^2^,^4^,^5^,^6^. Macrophages bearing a predominant M2 alternative activation phenotype are commonly found in deciduas at early pregnancy and have a central role in the ‘silent’ clearance of apoptotic cells^3^,^6^. Human trophoblast cells have been shown to favour such polarization with suppressor/regulatory signal induction^6^.

The vasoactive intestinal peptide (VIP) is a pleiotropic polypeptide with potent smooth muscle relaxing, vasodilating, pro-secretory and anti-inflammatory effects upon binding high affinity VPAC1 or VPAC2 receptors coupled to stimulatory G protein and adenylate cyclase activation and with lower affinity to PAC1 receptors^7^,^8^. VIP gene expression in human neuroblastoma cells is mediated by cAMP response element sites (CRE) and for gp130 family cytokines elements (CyRE) in its promoter^9^,^10^,^11^. Among gp130 family cytokines, the Leukemic inhibitory factor (LIF) has a relevant role in implantation and placentation processes^12^,^13^. VIP and VPAC2 receptor expression raise in the implantation sites at placentation between days 9,5 and 12,5 of murine pregnancy and VIP levels peak in serum at day 11,5 in rats^14^,^15^,^16^. Interestingly, VIP showed trophic effects on post-implantation mouse embryos explanted with their yolk sac at day 9,5 without inducing macroscopic abnormalities^17^, whereas VPAC receptor blockade reduced embryo weight gain and induced microcephaly associated with a thinner cortex area in mice^17^,^18^. Likewise, VIP treatment at day 6,5 of gestation of two resorption prone mouse models, the non obese diabetic mice and the CBA/J × DBA/2 mice, improved pregnancy outcome, increased the number of implanted embryos and the expression of alternatively activated macrophages and regulatory T cell markers^16^,^19^. In human pregnancy, VIP is expressed in cytotrophoblast and syncytiotrophoblast cells of first and third trimester placenta as well as in the third trimester trophoblast cell line JEG-3^20^. VIP high affinity receptors are expressed on JEG-3 cell line and VIP enhances hCG synthesis through cAMP response elements (CRE) in these cells^21^. Moreover, dose-dependent stimulation of progesterone release by VIP was also reported in JEG-3 cells and human trophoblast primary cultures^20^. VIP and VPAC receptors are also expressed in the human first trimester trophoblast cell line Swan 71^22^,^23^. VIP priming of two first trimester cell lines (Swan 71 and HTR8) enhances the phagocytosis of apoptotic cells by macrophages through thrombospondin-1/αvβ3 portal formation^24^. So far, there are no reports on VIP effects on migration and invasion capacities of human first trimester trophoblast cells, the signalling cascades and potential autocrine regulatory pathways involved. Here we explored the mechanisms of VIP synthesized by two human first trimester trophoblast cell lines on their invasion and migration capacity at the cellular and molecular level. We evaluated as well, its ability to enhance the clearance of apoptotic bodies and to induce an alternative activation profile on maternal macrophages. Our results demonstrate that VIP synthesized by human first trimester trophoblast cell lines Swan 71 and HTR8 increases cell migration and invasiveness involving PKA/CRE signalling and autocrine pathways. VPAC2 receptor over-expression mimicked VIP effects and VIP-silenced trophoblast cells displayed an impaired migration profile along with a reduced response to LIF. Finally, VIP knocked down trophoblast cells failed to promote the phagocytosis of apoptotic cells by human monocytes and to induce anti-inflammatory markers.

## Results

### VIP increases trophoblast cell migration through a PKA/CRE pathway

**Figure 1:**
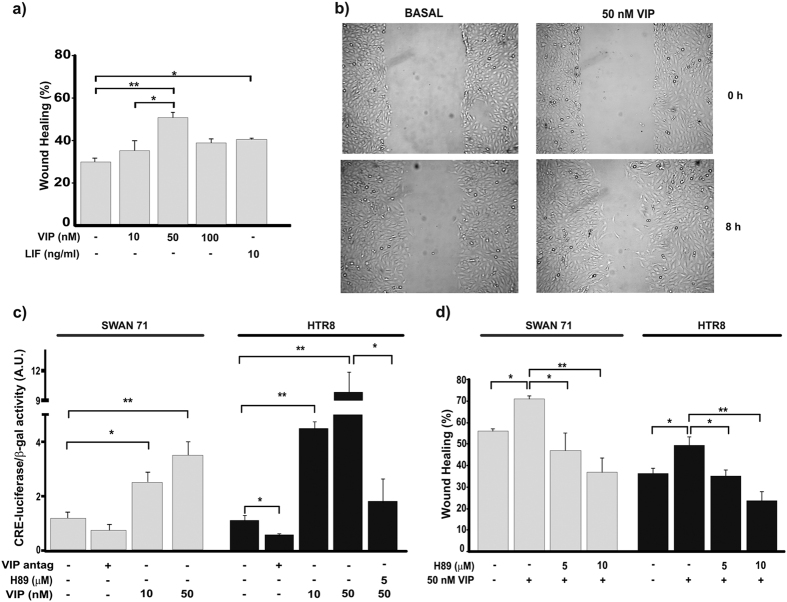
VIP increases trophoblast cell migration through PKA/CRE signalling. Trophoblast cells (Swan 71 cell line) were grown in medium supplemented with 10% FBS until they reached confluence and then wounded and incubated for 0–8 h in low serum medium without (Basal) or with VIP (10–100 nM) or 10 ng/ml LIF and photographed (AxioVision 4.0). The wound area was quantified with the same program. (**a**) Values given are expressed as mean ± S.E.M. (*P < 0.05; **P < 0.01; n = 4). (**b**) A representative experiment of 4 is shown. (**c**) Cells (Swan 71 and HTR8 lines) were co-transfected with a CRE-luciferase and a β-galactosidase plasmid for 24 h and then cells were incubated in the absence (Basal) or presence of VIP or 100 nM VIP antagonist for 24 h. When indicated, PKA activation was inhibited by pretreatment with 5 μM H89 for 30 min before the addition of VIP. CRE sites activation was measured by the luciferase activity reporter assay and results were normalized to β-galactosidase activity. The results are expressed as mean ± S.E.M. (*P < 0.05; **P < 0.01 n = 4). (**d**) Cells were grown in medium supplemented with 2% FBS until they reached confluence and then wounded and incubated for 0–16 h without (Basal) or with VIP in a low serum medium. When indicated, cells were pretreated with 5 or 10 μM H89. Values given are expressed as mean ± S.E.M. (*P < 0.05; **P < 0.01 n = 3).

Based on the role of trophoblast cell migration during placentation and considering that VIP binds VPAC receptors expressed on human first and third trimester trophoblast cell lines and increases cAMP and hormone synthesis^20^,^21^,^23^, we first explored VIP effect on migration of Swan 71 and HTR8 trophoblast cell lines and the involvement of PKA/CRE signalling cascade. In wound healing assays, we observed that VIP increased Swan 71 trophoblast cell migration in a dose dependent manner with a maximal effect at 50 nM (Fig. 1a,b). The effect was similar to that of 10 ng/ml LIF (Fig. 1a), a gp130 family cytokine involved in the placentation process^13^. To investigate the activation of PKA/CRE signalling by VIP on trophoblast cells, we performed transfection assays with a CRE-Luciferase reporter gene construction. First trimester trophoblast cell lines, Swan 71 and HTR8, showed a dose-dependent increase of luciferase activity in response to VIP indicating CRE sites activation in both cell lines (Fig. 1c). Moreover, when we pretreated the transfected cells with the PKA inhibitor H89 the effect of VIP on CRE sites activation was suppressed confirming the involvement of this kinase in VIP signaling (Fig. 1c). On the other hand, trophoblast cells transfected with CRE-Luc plasmid presented a diminished CRE activation when they were cultured in basal conditions in the presence of a VIP antagonist suggesting an autocrine activation of VIP signalling (Fig. 1c). To elucidate whether VIP induces migration through the activation of PKA, we performed wound healing assays in the presence of H89. As shown in Fig. 1d, the PKA inhibitor prevented the effect of 50 nM VIP on cell migration in both cell lines confirming the involvement of this pathway.

### VPAC2 receptor over-expression induces trophoblast cell migration and invasion

**Figure 2:**
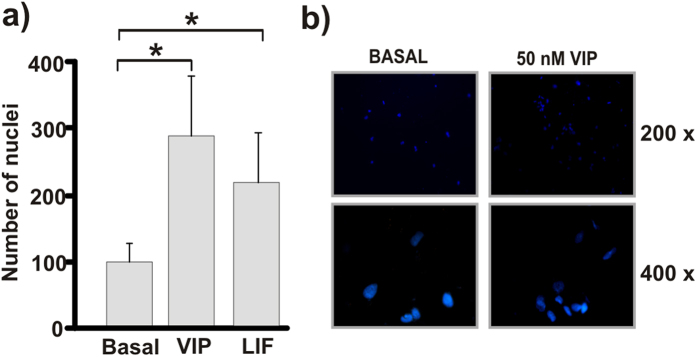
VIP promotes trophoblast cell invasion. Trophoblast cells (Swan 71 cell line) were resuspended in medium without (Basal) or with 50 nM VIP or 10 ng/ml LIF and then seeded on the upper chamber of *Matrigel*-coated transwells. After cell incubation for 40 h at 37 °C in 5% CO_2_ filters were washed and the cells in the lower surface were fixed and stained with DAPI. (**a**) The results are expressed as mean ± S.E.M. (*P < 0.05; n = 3). (**b**) A representative experiment of 3 with similar results is shown.

**Figure 3:**
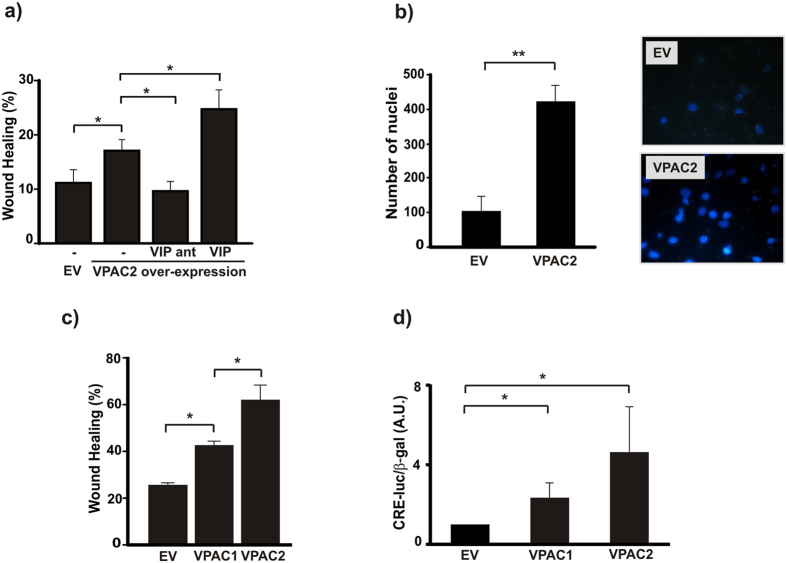
VPAC2 activation favours migration and invasion in a trophoblast-derived cell line. (**a**) Trophoblast cells (HTR8 cell line) were co-transfected with a VPAC2 plasmid to induce over-expression and a GFP plasmid to measure the transfection efficiency (see Fig. S2). 36 h post-transfection cells were subjected to wound healing assays in the absence (−) or presence of 50 nM VIP or 100 nM VIP antagonist for 8 h. The empty vector (EV) was used as negative transfection control (*P < 0.05; n = 3). (**b**) *Matrigel*-coated transwells were used to evaluate the invasive capability of the HTR8 cells after VPAC2 over-expression. 36 h post-transfection, trophoblast cells were gently seeded on the upper chamber of the matrigel coated transwell and incubated for 40 h at 37 °C in 5% CO2. Filters were washed and the cells on the lower surface were fixed and stained with DAPI. The results are expressed as mean ± S.E.M. (**P < 0.01; n = 4). Representative photographs of 4 experiments with similar results are shown. (**c**) Trophoblast cells were transfected with a VPAC1 or VPAC2 plasmid to induce over-expression and with CRE-Luc and β-gal at the same time. To evaluate cell migration, 36 h post transfection cells were subjected to wound healing assays for 16 h. Results are expressed as mean ± S.E.M. (*P < 0.05; n = 3). (**d**) 36 h post-tranfection CRE sites activation in trophoblast cells was measured as in Fig. 1. Results are expressed as mean ± S.E.M. (*P < 0.05; n = 3).

Invasion of the decidua by trophoblast cells is central to placentation so we determined the effect of VIP stimulation or VPAC2 receptor over-expression on first trimester trophoblast cells invasiveness. HTR8 cells were seeded on *transwells* coated with matrigel and incubated in the absence or presence of 50 nM VIP for 40 h. As shown in Fig. 2, there was a 3-fold increase of trophoblast invasion over the basal condition with 50 nM VIP. A comparable effect was observed when the cells were stimulated with 10 ng/ml LIF used as a positive control. On the basis that trophoblast cells express both VPAC1 and VPAC2 high affinity receptors, and that VPAC2 receptor expression is induced at high levels in uterus during murine pregnancy^16^, we next explored the effect of VPAC2 receptor over-expression on migration and invasion of HTR8 cells. Transfection assays carried out with a VPAC2 plasmid resulted in over-expression of this receptor with high efficiency as indicated by co-tranfection experiments with a green fluorescence protein (GFP) plasmid and by qRT-PCR (See Supplementary Fig. S1a,b). As shown in Fig. 3a, VPAC2 receptor over-expression increased trophoblast cell migration compared with the empty vector used as a control (EV). The effect was further increased with 50 nM VIP and inhibited in the presence of a VPAC receptor antagonist, suggesting an autocrine VIP contribution to the induction of VPAC2 receptor activation (Fig. 3a). On the other hand, *Matrigel* invasion assays showed an increased invasive capacity of HTR8 cells when VPAC2 receptor was over-expressed compared to the empty vector (Fig. 3b).

To investigate whether both VPAC1 and VPAC2 receptor subtypes were involved indistinctively in the modulation of trophoblast cell function, we analyzed cell migration in HTR8 cells over-expressing either VPAC1 or VPAC2 receptors. We found that VPAC1 receptor over-expression induces an increase of cell migration in HTR8 cells, however, the effect was lower than that observed in cells over-expressing VPAC2 receptor, suggesting a major VPAC2 contribution to cell migration (Fig. 3c). To explore whether this difference was related to a higher ability of VPAC2 over VPAC1 receptor subtype to induce PKA/CRE signalling, we co-transfected HTR8 cells with CRE-Luc, β-gal and either VPAC1 or VPAC2 plasmids and performed CRE-Luciferase activity reporter assays. As shown in Fig. 3d, both VPAC receptor subtypes effectively activated the PKA/CRE signalling to the same extent (Fig. 3d).

### VIP induces its own synthesis in first trimester trophoblast cell lines

**Figure 4:**
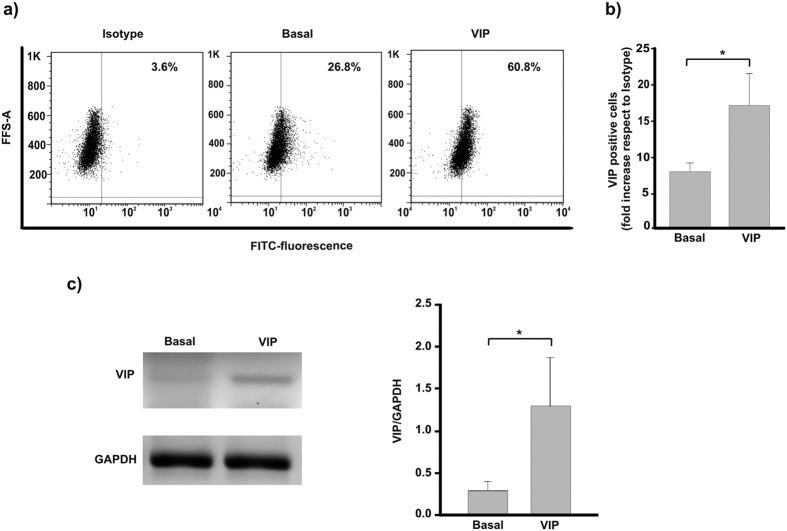
VIP induces its own synthesis in a first trimester trophoblast-derived cell line. Cells (Swan 71 cell line) were grown in 2% FBS medium in the absence (Basal) or presence of 10 nM VIP for 24 h. Intra-cytoplasmatic detection of VIP was quantified by flow cytometry. (**a**) Representative dot plots of 3 experiments are shown. (**b**) The percentage of positive cells are expressed as fold increase respect to isotype control and are the mean ± S.E.M. (*P < 0.05; n = 3). (**c**) Cells (Swan 71 cell line) were grown in 2% FBS medium in the absence (Basal) or presence of 100 nM VIP for 6 h and VIP mRNA levels assessed by RT-PCR as indicated in material and methods. Representative gels from 3 experiments and band densitometry using Image J program are shown (*P < 0.05; n = 3).

Based on previous reports that VIP gene expression is induced in human neuroblastoma cells through activation of CRE and CyRE sites in its promoter^9^,^10^,^11^,^21^, and that VIP and VPAC receptors are expressed on human first and third trimester trophoblast cells^20^,^23^, we next explored if exogenous VIP could induce its own synthesis in Swan 71 first trimester trophoblast cells. Swan 71 cells cultured in the presence of 10 nM VIP for 24 h increased the expression of VIP as detected by flow cytometry and RT-PCR (Fig. 4).

### VIP knock-down decreases basal and LIF-induced trophoblast cell migration

**Figure 5:**
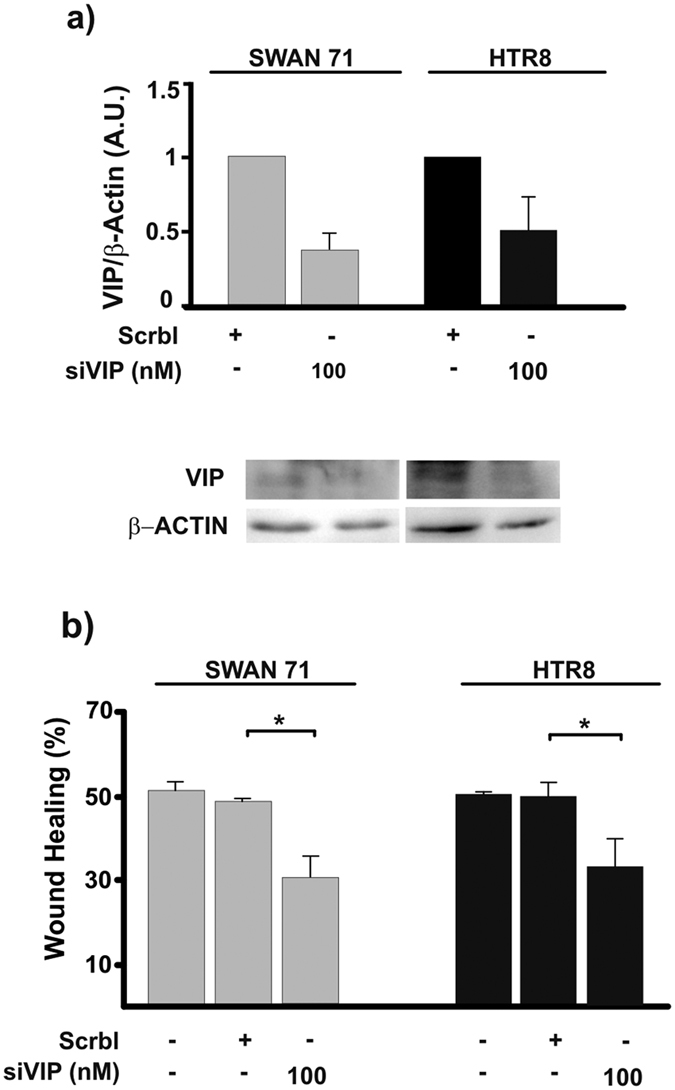
VIP silencing in trophoblast cells decreases basal cell migration. (**a**) First trimester trophoblast cells (Swan 71, HTR8 cell lines) were transfected with 100 nM VIP siRNA (siVIP) or a siRNA with a scramble sequence as control (Scrbl) for 24 h, washed with warm medium and incubated for other 48 h in fresh 10% FBS medium. (**a**) Total protein samples were subjected to western blotting to confirm the protein knock down. The image shows cropped lines corresponding to β-actin and VIP coming from the same blot. Blots were run under the same experimental conditions. (**b**) 72 h post transfection cells were subjected to wound healing assays to evaluate cellular migration for 16 h. Results are expressed as mean ± S.E.M. (*P < 0.05; n = 4).

Following the observation that the antagonist of VIP receptors reduced CRE signalling in non-stimulated trophoblast cells (Fig. 1c) as well as cell migration in VPAC2 receptor over-expressing cells (Fig. 3a), we carried out loss of function experiments to investigate the relevance of endogenous VIP in first trimester trophoblast cell function. We performed knocking down experiments using a VIP siRNA in HTR8 and Swan 71 cells for 72 h and, after confirming the decrease of VIP expression (Fig. 5a), wound healing assays were carried out to evaluate cell migration. The results shown in Fig. 5b indicate a significant inhibition of trophoblast cell migration in both cell lines in basal conditions, confirming the autocrine effect of VIP in this process.

**Figure 6:**
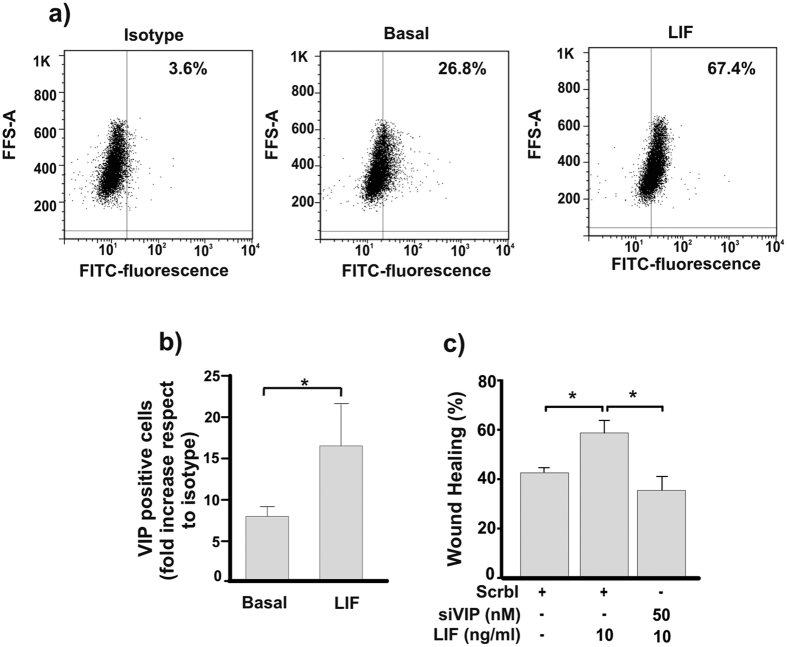
Endogenous VIP is involved in trophoblast cell migration induced by LIF. (**a**) VIP expression was determined as in Fig. 4 in Swan 71 cells cultured with 10 ng/ml LIF. Representative dot plots of 3 experiments with similar results were shown. (**b**) Percentage of positive cells is expressed as fold increase respect to isotype control and are the mean ± S.E.M (*P < 0.05, n = 3). (**c**) 72 h post-transfection with Scrbl siRNA or VIP siRNA, Swan 71 cells were subjected to wound healing assays in the absence or presence of 10 ng/ml LIF. Results are expressed as mean ± S.E.M. (*P < 0.05; n = 3).

It is well known that LIF promotes trophoblast cell migration and invasion^12^,^13^,^25^. On this basis and taking into account the effect of VIP on trophoblast cells, we explored whether the effect of LIF on migration could be mediated by VIP. First, we determined if LIF could induce the synthesis of VIP in first trimester trophoblast cells. Figure 6a,b shows that LIF (10 ng/ml) increased the synthesis of VIP in trophoblast cells. Since Swan 71 cells treated with 10 ng/ml LIF showed an increment of cell migration after 8 h of culture (Fig. 1a), we next investigated whether VIP is involved in trophoblast cell migration induced by LIF. Wound healing assays were performed with VIP silenced Swan 71 cells cultured in the presence of LIF. The increase of cell migration induced by LIF in basal or scramble siRNA transfected (control) cells was not observed in VIP silenced cells, suggesting that endogenous VIP participates in trophoblast cell migration induced by LIF (Fig. 6b).

### VIP knock-down in trophoblast cell lines impairs immunosuppressant phagocytosis of apoptotic cells

**Figure 7:**
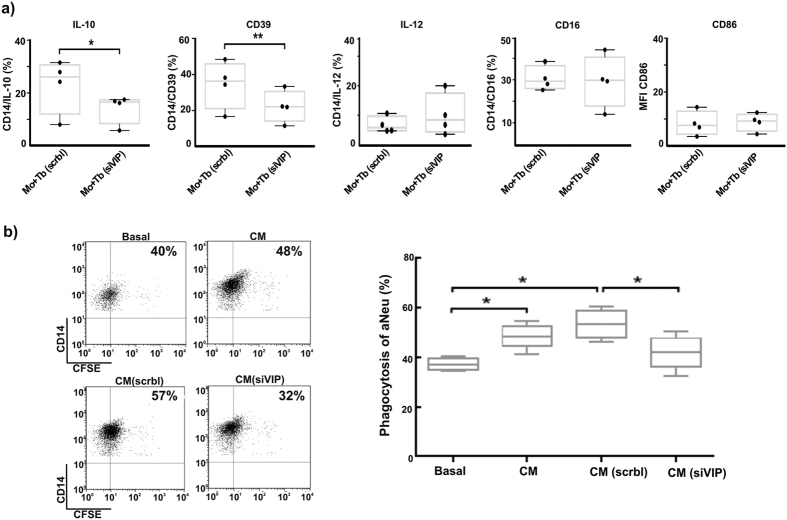
VIP produced by trophoblast cells induces an anti-inflammatory activation profile of monocytes. Human purified monocytes were plated and co-cultured with Swan 71 cells transfected with VIP siRNA or Scrbl siRNA as described in Material and Methods. (**a**) CD14 positive cells activation profile was analyzed by flow citometry. MFI of CD86 and the percentage of double positive CD14+/IL-10+ or IL-12+ or CD39+ or CD16+ values are expressed as mean ± S.E.M. of 3 experiments with different human monocyte samples (*P < 0.05; *P < 0.01; n = 4; Mann Whitney). (**b**) Monocytes were incubated with conditioned media from scrbl (CM scrbl) or VIP siRNA (CM siVIP) transfected or non-trasnfected (CM) trophoblast cells for 20 h and then challenged with CFSE+ apoptotic neutrophils during 40 min and the percentage of CD14/CFSE positive cells analyzed by FACS. A representative dot plot of double positive cells is shown (Left panel). Results are expressed as mean ± S.E.M. (*P < 0.05; n = 4; Mann Whitney) (Right panel).

We have recently demonstrated that VIP primes Swan 71 cells to promote an anti-inflammatory profile on human monocytes and macrophages and enhance apoptotic cell phagocytosis^26^. On this basis, we next explored whether VIP produced by trophoblast cells could be involved in monocyte/macrophage modulation. VIP-silenced Swan 71 cells were co-cultured with monocytes and their phenotype and function were assessed. We analyzed the expression of monocyte anti-inflammatory (IL-10, CD39) and pro-inflammatory (IL-12, CD86 and CD16) markers after the co-cultures. The results shown in Fig. 7a indicate an impaired ability of VIP-knocked down Swan 71 trophoblast cells to induce an anti-inflammatory profile on monocytes compared with scramble siRNA-transfected cells. To determine whether VIP produced by trophoblast cells also modulates phagocytosis of apoptotic cells by monocytes, phagocytosis assays were carried out after incubating monocytes with trophoblast conditioned media (CM). Figure 7b shows that conditioned media from VIP siRNA transfected Swan 71 cells was not effective to increase apoptotic cell phagocytosis by monocytes compared with the conditioned media from scramble siRNA transfected control cells, supporting the relevance of VIP produced by these trophoblast cells in the modulation of phagocytosis and the induction of an immunosuppressant microenvironment.

## Discussion

The generation of the human maternal-placental interface requires trophoblast differentiation into phenotypes able to migrate and invade the decidua. Impaired invasiveness and the loss of immune homeostasis associate with placental insufficiency and pregnancy complications as preeclampsia and IUGR^1^,^3^,^27^. Since VIP is a pleiotropic polypeptide with trophic and immunomodulatory effects in different pregnancy models, we investigated VIP autocrine pathways that regulate trophoblast function through high affinity VPAC1 and VPAC2 receptors and their interaction with phagocytic cells by means of loss and gain of function assays performed in two first trimester trophoblast cell lines.

Evidence presented here indicates that VIP promotes first trimester trophoblast cell migration through PKA/CRE signalling pathways involving both VPAC1 and VPAC2 receptor subtypes. VIP acts through autocrine pathways to regulate basal and LIF-induced trophoblast migration as well as to enhance apoptotic cell phagocytosis by monocytes expressing an immunosuppressant profile. These conclusions are based on the following observations: First, VIP dose-dependently increased trophoblast cell migration and invasion. VIP increased PKA/CRE signalling in a dose-dependent manner, which was blocked in the presence of a PKA inhibitor. Furthermore, cells transfected with a reporter CRE-Luc plasmid showed a decrease of PKA/CRE signalling in the presence of VIP receptor antagonist suggesting the activation of this pathway by endogenous VIP in basal conditions. Second, VPAC receptor over-expression increased trophoblast cell migration with a major contribution of VPAC2 receptor subtype although similar CRE sites activation levels were associated to VPAC2 and VPAC1 receptor over-expression. Third, VIP induced its own synthesis in first trimester trophoblast cells and VIP silencing reduced basal and LIF-mediated trophoblast cell migration. Finally, VIP knocked-down trophoblast cells failed to induce IL-10 and CD39 expression on monocytes and to enhance apoptotic cell phagocytosis by these cells.

The expression of VIP, VPAC1 and VPAC2 receptors was demonstrated on first trimester trophoblast cells^20^,^23^ and VPAC2 receptors seem to be particularly involved at early stages of murine pregnancy characterized by high progesterone levels^16^,^28^. Besides, VIP is induced in human neuroblastoma cells through the activation of CRE and CyRE sites in its promoter by either hCG or LIF^9^,^10^,^11^. Our results indicate that these pathways are active in two human first trimester trophoblast cell lines and that they are involved in certain functional capacities central to the placentation process such as migration, invasion and the response to LIF. Moreover, the observation that a VIP receptor antagonist decreased trophoblast cell migration in VPAC2 receptor over-expressing cells respect to basal conditions also supports a role for endogenous VIP on VPAC2 receptor activation through PKA/CRE signalling. In this regard, the relevance of endogenous VIP was confirmed after VIP knocking down experiments where the decrease observed in trophoblast cell migration strongly supports the involvement of VIP regulatory autocrine mechanisms in the first trimester trophoblast cells studied.

LIF is a gp130 family cytokine essential for implantation and placentation processes in mice and its role in human trophoblast cell invasion has been demonstrated^12^,^13^. Interestingly, here we demonstrated that the migration induced by VIP in two human first trimester trophoblast cell lines involves VIP synthesis since its effect was blocked in VIP silenced cells.

Finally, we have recently reported that VIP primes human trophoblast cells to increase apoptotic cell phagocytosis by monocytes and macrophages along with their polarization to an anti-inflammatory phenotype^24^. Results presented here confirm and extend this observation pointing to the first trimester trophoblast cell as an endogenous source of the neuropeptide. The modulatory effect of trophoblast cells on monocytes and macrophages phenotype and function at the maternal-fetal interface has been supported by many *in vitro* and *in vivo* observations^6^,^29^,^30^,^31^,^32^. Human trophoblast cells enhance macrophage polarization to a predominant M2 alternative activation phenotype which is the most common phenotype found at early placentation providing suppressor/regulatory signals^6^,^30^,^32^. In preeclampsia, macrophages express an M1 predominant phenotype whereas their localization at the maternal-fetal interface is altered^6^. In line with these observations, our results support that one of the locally synthesized mediators at the close interaction between trophoblast cells and macrophages is VIP produced by trophoblast cells. Moreover, our results propose that upon activation of VPAC receptors, not only basal levels of VIP but also VIP-induced VIP levels in trophoblast cells would contribute to autocrine/paracrine regulation of trophoblast migration and the immunosuppressant clearance of apoptotic bodies at early stages of pregnancy.

Pituitary adenylate cyclase activating polypeptide (PACAP) is another member of the VIP/secretin/glucagon family that activates both VPAC and PAC1 receptors and is expressed in human placenta^33^. Interestingly, PACAP-38 was not effective to enhance HTR8 cell line invasiveness^34^, supporting the specific role of VIP to modulate first trimester trophoblast cell line function. However, since both neuropeptides can activate VPAC receptors with similar affinities in other cell types^8^ these differences might also be related to the experimental conditions, particularly the shorter incubation time to assess invasiveness (40 h vs. 72 h) used here. Moreover, on the basis that PACAP but not VIP binds PAC1 receptors with high affinity, these differences would also confirm the specificity of VPAC receptors for the effects on this human trophoblast cell line. Another interesting difference between PACAP-38 and VIP is related to their ability to cross the plasma membrane in a receptor-independent manner and activate intracellular receptors^35^. Eventhough both peptides contain basic residues, PACAP38 has larger positive charge and displayed almost ten fold higher uptake by CHO cells compared with a modest uptake of VIP and other secretin/glucagon family members^36^. Moreover, it was proposed that once inside the cells, PACAP38 binds to nuclear fractions from various rat tissues where PACAP but not VIP appeared to activate PAC1 intracellular receptors^35^.

Defective transformation of uterine spiral arteries by trophoblast cells with a persistent pro-inflammatory response were shown as predisposing factors for deep placentation associated with high rates of maternal and fetal morbidity and mortality^2^. Triggers of this abnormal inflammatory process are poorly understood and the role of a defective trophoblast invasion and interaction with immune and decidual cells has been proposed. Whereas in normal human pregnancies enhanced apoptosis of endothelial and trophoblast cells is followed by an immediate removal of apoptotic bodies by professional phagocytes in an immunosuppressant microenvironment, an imbalance of trophoblast apoptosis and pro-inflammatory processes was described in pre-term labor, IUGR and PE^1^,^3^,^27^,^32^.

Our results suggest that VIP-induced VIP synthesis by trophoblast cells might be one of the putative mechanisms that regulate trophoblast cell migration and invasion *in vivo*, as well as their interaction with phagocytic cells to contribute to immune homeostasis. These observations provide new evidence on autocrine VIP-mediated regulatory pathways that might be active during normal placentation and which impairment could underlie the aetiology of pregnancy complications associated with defective deep placentation.

## Material and Methods

### Trophoblast-derived cell line cultures

Two trophoblast cell lines from human first trimester pregnancies were used throughout. HTR-8/SVneo cell line (HTR8) was derived from transformed extravillous trophoblast and Swan 71 cell line by telomerase-mediated transformation of a 7-week cytotrophoblast isolate were kindly given by Dr Gil Mor (Yale University, New Haven, USA)^37^,^38^. Cells were maintained in culture flasks at 37 °C, 5% CO_2_ in Dulbeco’s modified Eagle’s medium and Nutrient Mixture F-12 (DMEM-F12) (Life Technologies, Grand Islands, NY, USA) containing 25 mM HEPES and supplemented with 10% heat-inactivated fetal bovine serum (FBS), 2 mM Glutamine (Sigma-Aldrich) and 100 U/ml streptomycin-100 μg/ml penicillin solution (Life Technologies, Grand Islands, NY, USA).

### VIP expression

Trophoblast cell lines were incubated with Stop Golgi (Becton Dickinson, San José, CA) for the last 4 h of cell culture following manufacturer’s instructions to promote intracellular accumulation of proteins. After washing with PBS-2% FBS (staining buffer), cells were fixed and permeabilized with the Fix/Perm kit according to manufacturer’s instructions (Becton Dickinson, San José, CA). Permeabilized cells were incubated for 30 min with human VIP monoclonal antibody at room temperature. Cells were washed with staining buffer twice and then stained with a secondary antibody Alexa 488 for 45 min. Ten thousand events were acquired in a FACS Aria II cytometer^®^ (Becton Dickinson, San José, CA, USA) and the data was analyzed using the FlowJo software (http://www.flowjo.com/). Results were expressed as fold increase of VIP positive cells respect to the isotype control.

### Cell migration assay

Wound healing assays were carried out to evaluate trophoblast migration. Briefly, 4 × 10^4^ cells were plated in 24-well polystyrene plates with DMEM-F12 10% FBS and incubated in humidified chamber with 5% CO_2_ at 37 °C. When cells reached confluence, a wound was made with a sterile tip and the monolayer was washed to eliminate unattached cells. Photographs were taken at different times (0–24 h) and the images were analyzed using the AxioVision program. Results were expressed as the percentage of wound healing analyzing the whole wound area.

### Cell invasion assay

To evaluate trophoblast invasiveness, 5 × 10^4^ cells were re-suspended in DMEM-F12 without or with the stimuli and seeded on the upper chamber of a 6.5 mm *Transwell* with 8.0 μm pore polycarbonate membrane insert pre-coated with 30 μl of reduced-growth factors *Matrigel*, Geltrex (Life Technologies, Grand Islands, NY, USA). After 24–40 h of incubation, the transwell inserts were washed and the lower surface was fixed and stained with DAPI. This two chamber system assesses both cell invasion and migration. Photographs were taken and nucleus counted in 10 randomly non-overlapping fields.

### Plasmid transfection of trophoblast cells

Transfection assays were performed using the X-tremeGENE HP DNA (Roche, Mannheim, Germany) reagent and the corresponding plasmids (CDNA Resource Centre Bloomsburg University, USA) following manufacturer’s instructions. Briefly, plasmids and the transfection reagent diluted in Optimem^®^ (Life Technologies, Grand Islands, NY, USA) in a 1:1 ratio (μg DNA: μl reagent) were mixed gently and incubated for 15 min at room temperature. The transfection reagent:plasmid complex were added drop wise to the cells. After 24 h, the media was renewed and the stimuli were added. Wound healing assays were carried out at this point to evaluate the effect of VPAC1 or VPAC2 receptor over-expression compared to Empty Vector (EV, pCDNA 3.1+) transfection. Transfection efficiency was determined by co-transfecting with an expression vector of green fluorescence protein (GFP) and quantifying de number of fluorescent cells in 10 randomly selected fields. We obtained a 65% transfection efficency. To confirm VPAC2 receptor over-expression, mRNAs were analyzed by qRT-PCR as described below. To evaluate the CRE sites activation, both trophoblast cell lines (Swan 71 and HTR8) were co-transfected with 6× CRE-Luciferase and β-galactosidase plasmids for 24 h as described above, and then after media renewal cells were cultured with the stimuli for other 24 h. CRE sites activation was determined by the luciferase activity reporter assay (Promega Corporation, Madison, WI, USA) following manufacturer’s instructions. Briefly, attached cells were rinsed with PBS, covered with lysis buffer and scraped from the plate. The liquid was transferred to microcentrifuge tubes, vortexed and then centrifuged at 12000 × g for 2 minutes at 4 °C. The supernatant was transferred and 20 μl of cell lysates were mixed with 30 μl of Luciferase assay reagent placed into a 96 multiwell plate and read immediately in the luminometer. The results were normalized to β-galactosidase activity measured by spectrophotometry in cell lysates incubated with a reaction buffer containing ONPG.

### qRT-PCR

VIP receptors messenger RNA (mRNA) expression was analyzed by quantitative reverse transcription–polymerase chain reaction (qRT-PCR) as previously described^22^,^34^. Total RNA (1 μg) was treated with DNAasa I following manufacturer’s instructions (Sigma-Aldrich, San Luis, MO, USA) to avoid DNA contamination and then samples were reverse transcribed using a MMLV reverse transcriptase, RNAse inhibitor and oligodT kit (Promega Corporation, Madison, WI, USA) and stored at −20 °C for batch analysis. Samples were incubated with SYBR Green PCR Master Mix and the forward and reverse primers. The qRT-PCR was performed on a Bio-Rad iQ5 Real-time PCR system. The relative gene expression levels were determined using the threshold cycle (CT) method (2^−∆∆CT^ method) normalized to the endogenous GAPDH gene control. VIP RT-PCR assays, primers sequences and cycling conditions were the same as in^22^,^39^.

### VIP silencing

To transfect Swan 71 cells with a VIP siRNA (Santa Cruz Biotechnology, Dallas, TX, USA), cells were grown at 60% of confluence and arrested for 3 h in Optimem^®^. 50–100 nM VIP siRNA: Lipofectamine RNAimax (Life Technologies, Grand Island, NY, USA) complex were made in Optimem and incubated for 15 min prior to addition to the cells in a drop wise manner. 24 h post-transfection, the media were changed for DMEM-F12 supplemented with 10% FBS for other 48 h prior to the experiments. siRNA with a scramble sequence was used as a negative control (Scrbl).

### Western blotting

For protein extraction, cells were rinsed with PBS and scraped with 100 μl of 2X Lysis & Loading Buffer (200 mM Tris-HCl, pH 6.8; 8% SDS; 0.4% w/v Blue Bromophenol; 40% glycerol; 400 mM β-mercaptoethanol) and boiled for 4 min. Samples were subjected to SDS-PAGE electrophoresis and electro-blotted onto a nitrocellulose (NC) membrane during 1.15 h (transfer buffer: 25 mM Tris, 195 mM glycine, 0.05% SDS, pH 8.3, and 20% (v/v) methanol). Membranes were blocked by 1 h-incubation in Tris buffer saline (25 mM Tris, 137 mM NaCl, 3 mM KCl, pH 7.4) containing 0.1% Tween 20 and 0.5% skim-milk powder. Then, NC membranes were incubated with anti human VIP mAb (Santa Cruz Biotechnology, Dallas, TX, USA) over night at 4 °C. After washing with TBS–0.1% Tween 20, the NC were incubated with anti-mouse horseradish peroxidase-conjugated antibody (1:1,000) for 1 h at 20 °C and washed. Specific antibody signals were detected using the enhanced Chemiluminescence system ECL Plus kit (GE Healthcare, United Kingdom) and Fujifilm Intelligent Dark Box II equipment (Fuji) coupled to a LAS-1000 digital camera.

### Trophoblast cell lines conditioned media

To obtain conditioned media (CM), Swan 71 cells were transfected with a VIP siRNA or a Scrbl siRNA as negative control for 72 h as above in 24-well flat-bottom polystyrene plates with DMEM:F12 10% FBS. CM from Swan 71 non-transfected cells were used as control. Trophoblast cell CM were collected and stored at −80 °C until used.

### Monocyte isolation

Monocytes were isolated from blood samples drawn from healthy women volunteers at reproductive age who were not under pharmacological treatment for at least 10 days before the day of sampling. Blood was obtained by puncture of the forearm vein, and it was drawn directly into heparin containing plastic tubes. Studies were approved by the Argentine Society of Clinical Investigation Board and Ethical Committee (Ref. SAIC 46/14). All healthy donors provided written informed consent for sample collection and subsequent analysis. The methods were carried out in accordance with the approved guidelines. Peripheral blood mononuclear cells (PBMC) were processed from individual subjects by Ficoll-Hypaque and CD14+ cells separated by Percoll gradient (GE Healthcare, Sweden) as manufacturer’s protocol. Cell population purity (>80%) was checked by flow cytometry analysis with CD14 labeling as previously^39^.

### Monocyte pro and anti-inflammatory marker assessment

Monocytes were co-cultured with VIP siRNA transfected Swan 71 cells or Srcbl siRNA transfected cells as control, and after 20 hours cells were recovered by TrypLe^TM^ treatment (Life technologies, Grand Island, NY, USA) and stained with FITC, PE-Cy5, APC or PE-conjugated mAbs directed to surfer markers (CD14, CD86, CD39, CD16) or intracellular cytokines (IL-10, IL-12) (BD Pharmingen, San Diego, CA, USA). For intracellular cytokine detection Stop Golgi was added to the medium in the last 4 h of co-culture following manufacturer’s instructions to promote intracellular accumulation. Then cells were recovered and, after washing with staining buffer, they were stained with mAb anti-superficial molecules, washed, fixed and permeabilised with the Fix/Perm kit as manufacturer recommended. Permeabilised cells were incubated for 30 min with IL-10 or IL-12. Cells were finally washed with PBS-2% FBS. Ten thousand events were acquired in a FACS Aria II cytometer^®^ (Becton Dickinson, San José, CA, USA) results were analyzed using FlowJo software (http://www.flowjo.com/) expressed as MFI or double positive cell frequencies by flow cytometry. For monocyte profile, positive cells were determined inside the electronically gated CD14 positive cell population previously selected in FSC vs. SSC as previously^39^.

### Phagocytosis of apoptotic neutrophils by monocytes

Neutrophils were obtained after Ficoll-Hypaque gradients and subsequent Dextran purification^40^,^41^. Apoptotic neutrophils were obtained by spontaneous apoptosis after 16 h incubation in RPMI 1640 and stained with CFSE as above. The percentage of apoptosis was 50% for neutrophils as determined by annexin-propidium iodide staining and flow cytometry detection in a FACS Aria II cytometer^®^ (Becton Dickinson, San José, CA, USA). Results were analyzed using FlowJo software (http://www.flowjo.com/).

After isolation, monocytes (5 × 10^5^) were cultured in 24-well polystyrene plates and conditioned for 20 h with DMEM:F12 10% FBS, CM from Swan 71 cells non-transfected or transfected with a VIP siRNA (CM siVIP) or with a scrbl siRNA (CM scrbl) as control. Then, monocytes were challenged with apoptotic neutrophils stained with CFSE in a 10:1 ratio with respect to monocytes. After 40 min of phagocytosis, monocytes were collected, stained with CD14 and the percentage of CD14/CFSE double positive cells was analyzed by flow cytometry as described^39^.

### Statistical analysis

The significance of the results was analyzed by Student’s t test or Mann-Whitney test for parametric or nonparametric samples. When multiple comparisons were necessary ANOVA of two way factors or the Wilcoxon test were used with post-hoc tests Bonferroni or Holm-Sidak. Differences between groups were considered significant at P < 0.05 using the GraphPad Prism4 software (GraphPad, San Diego, CA, USA).

## Additional Information

**How to cite this article**: Vota, D. *et al*. Vasoactive Intestinal Peptide modulates trophoblast-derived cell line function and interaction with phagocytic cells through autocrine pathways. *Sci. Rep*. **6**, 26364; doi: 10.1038/srep26364 (2016).

## Supplementary Material

Supplementary Information

## References

[b1] Huppertz B., Weiss G. & Moser G. Trophoblast invasion and oxygenation of the placenta: measurements versus presumptions. J. Reprod. Immunol. 101–102, 74–79 (2014). 10.1016/j.jri.2013.04.003 23747129

[b2] Khong Y. & Brosens I. Defective deep placentation. Best Pract. Res. Clin. Obstet. Gynaecol. 25, 301–11 (2011). 21109492 10.1016/j.bpobgyn.2010.10.012

[b3] Mor G. & Cardenas I. The immune system in pregnancy: a unique complexity. Am. J. Reprod. Immunol. 63, 425–433 (2010). 20367629 10.1111/j.1600-0897.2010.00836.x PMC3025805

[b4] Ball E., Bulmer J. N., Ayis S., Lyall F. & Robson S. C. Late sporadic miscarriage is associated with abnormalities in spiral artery transformation and trophoblast invasion. J. Pathol. 208, 535–42 (2006). 16402350 10.1002/path.1927

[b5] Plaks V. . Matrix metalloproteinase-9 deficiency phenocopies features of preeclampsia and intrauterine growth restriction. Proc. Natl. Acad. Sci. USA 110, 11109–14 (2013). 23776237 10.1073/pnas.1309561110 PMC3704020

[b6] Fest S. . Trophoblast-macrophage interactions: a regulatory network for the protection of pregnancy. Am. J. Reprod. Immunol. 57, 55–66 (2007). 17156192 10.1111/j.1600-0897.2006.00446.x

[b7] Waschek J. A. VIP and PACAP: neuropeptide modulators of CNS inflammation, injury, and repair. Br. J. Pharmacol. 169, 512–23 (2013). 23517078 10.1111/bph.12181 PMC3682700

[b8] Couvineau A. & Laburthe M. VPAC receptors: structure, molecular pharmacology and interaction with accessory proteins. Br. J. Pharmacol. 166, 42–50 (2012). 21951273 10.1111/j.1476-5381.2011.01676.x PMC3415636

[b9] Symes A. . STAT proteins participate in the regulation of the vasoactive intestinal peptide gene by the ciliary neurotrophic factor family of cytokines. Mol. Endocrinol. 8, 1750–63 (1994). 7708062 10.1210/mend.8.12.7708062

[b10] Hahm S. H. & Eiden L. E. Two separate cis-active elements of the vasoactive intestinal peptide gene mediate constitutive and inducible transcription by binding different sets of AP-1 proteins. J. Biol. Chem. 274, 25588–93 (1999). 10464293 10.1074/jbc.274.36.25588

[b11] Jones E. A., Conover J. & Symes A. J. Identification of a novel gp130-responsive site in the vasoactive intestinal peptide cytokine response element. J. Biol. Chem. 275, 36013–20 (2000). 10964933 10.1074/jbc.M007373200

[b12] Vogiagis D. & Salamonsen L. A. Review: The role of leukaemia inhibitory factor in the establishment of pregnancy. J. Endocrinol. 160, 181–90 (1999). 9924186 10.1677/joe.0.1600181

[b13] Poehlmann T. G. . Trophoblast invasion: tuning through LIF, signalling via Stat3. Placenta 26 Suppl A, S37–41 (2005). 15837065 10.1016/j.placenta.2005.01.007

[b14] Hill J. M. . Maternal vasoactive intestinal peptide and the regulation of embryonic growth in the rodent. J. Clin. Invest. 97, 202–208 (1996). 8550835 10.1172/JCI118391 PMC507080

[b15] Spong C. Y. . Maternal regulation of embryonic growth: the role of vasoactive intestinal peptide. Endocrinology 140, 917–924 (1999). 9927324 10.1210/endo.140.2.6481

[b16] Hauk V. . Vasoactive intestinal Peptide induces an immunosuppressant microenvironment in the maternal-fetal interface of non-obese diabetic mice and improves early pregnancy outcome. Am. J. Reprod. Immunol. 71, 120–130 (2014). 24405265 10.1111/aji.12167

[b17] Gressens P., Hill J. M., Gozes I., Fridkin M. & Brenneman D. E. Growth factor function of vasoactive intestinal peptide in whole cultured mouse embryos. Nature 362, 155–8 (1993). 8383805 10.1038/362155a0

[b18] Passemard S. . VIP blockade leads to microcephaly in mice via disruption of Mcph1-Chk1 signaling. J. Clin. Invest. 121, 3071–87 (2011). 21737879 10.1172/JCI43824 PMC3148726

[b19] Gallino L. . VIP treatment prevents embryo resorption by modulating efferocytosis and activation profile of maternal macrophages in the CBAxDBA resorption prone model prone model. Sci. Rep. doi: 10.1038/srep18633 (2016). PMC4702085 26733206

[b20] Marzioni D. . Placental expression of substance P and vasoactive intestinal peptide: evidence for a local effect on hormone release. J. Clin. Endocrinol. Metab. 90, 2378–2383 (2005). 15623814 10.1210/jc.2004-1512

[b21] Deutsch P. J., Sun Y. & Kroog G. S. Vasoactive intestinal peptide increases intracellular cAMP and gonadotropin-alpha gene activity in JEG-3 syncytial trophoblasts. Constraints posed by desensitization. J. Biol. Chem. 265, 10274–10281 (1990). 1693918

[b22] Fraccaroli L. . VIP modulates the pro-inflammatory maternal response, inducing tolerance to trophoblast cells. Br. J. Pharmacol. 156, 116–126 (2009). 19133995 10.1111/j.1476-5381.2008.00055.x PMC2697780

[b23] Fraccaroli L. . VIP boosts regulatory T cell induction by trophoblast cells in an *in vitro* model of trophoblast-maternal leukocyte interaction. J. Leukoc. Biol. 98, 49–58 (2015). 25877932 10.1189/jlb.1A1014-492RR PMC6608015

[b24] Paparini D. . VIP enhances apoptotic cell phagocytosis by monocyte/macrophages in an *in vitro* model of immune-trophoblast interaction. Placenta 36, 495 (2015).

[b25] Tapia A., Salamonsen L. A., Manuelpillai U. & Dimitriadis E. Leukemia inhibitory factor promotes human first trimester extravillous trophoblast adhesion to extracellular matrix and secretion of tissue inhibitor of metalloproteinases-1 and -2. 23, 1724–1732 (2008). 10.1093/humrep/den121 PMC2474668 18492704

[b26] Paparini D. . Trophoblast cells primed with vasoactive intestinal peptide enhance monocyte migration and apoptotic cell clearance through αvβ3 integrin portal formation in a model of maternal-placental interaction. Mol. Hum. Reprod. 21, 930–41 (2015). 26502804 10.1093/molehr/gav059

[b27] Redman C. W. G. & Sargent I. L. Immunology of pre-eclampsia. Am. J. Reprod. Immunol. 63, 534–543 (2010). 20331588 10.1111/j.1600-0897.2010.00831.x

[b28] Roca V. . Potential immunomodulatory role of VIP in the implantation sites of prediabetic nonobese diabetic mice. Reproduction 138, 733–742 (2009). 19633131 10.1530/REP-09-0171

[b29] Grasso E. . Differential migration and activation profile of monocytes after trophoblast interaction. PLoS One 9, e97147 (2014). 24849800 10.1371/journal.pone.0097147 PMC4029600

[b30] Abrahams V. M., Kim Y. M., Straszewski S. L., Romero R. & Mor G. Macrophages and apoptotic cell clearance during pregnancy. Am. J. Reprod. Immunol. 51, 275–282 (2004). 15212680 10.1111/j.1600-0897.2004.00156.x

[b31] Straszewski-Chavez S. L., Abrahams V. M. & Mor G. The role of apoptosis in the regulation of trophoblast survival and differentiation during pregnancy. Endocr. Rev. 26, 877–897 (2005). 15901666 10.1210/er.2005-0003

[b32] Nagamatsu T. & Schust D. J. The immunomodulatory roles of macrophages at the maternal-fetal interface. Reprod. Sci. 17, 209–218 (2010). 20065301 10.1177/1933719109349962

[b33] Brubel R. . Changes in the expression of pituitary adenylate cyclase-activating polypeptide in the human placenta during pregnancy and its effects on the survival of JAR choriocarcinoma cells. J. Mol. Neurosci. 42, 450–8 (2010). 20449689 10.1007/s12031-010-9374-5

[b34] Horvath G. . Investigation of the possible functions of PACAP in human trophoblast cells. J. Mol. Neurosci. 54, 320–30 (2014). 24874580 10.1007/s12031-014-0337-0

[b35] Doan N.-D. . Receptor-independent cellular uptake of pituitary adenylate cyclase-activating polypeptide. Biochim. Biophys. Acta 1823, 940–9 (2012). 22343001 10.1016/j.bbamcr.2012.02.001

[b36] Neree A. T., Nguyen P. T. & Bourgault S. Cell-Penetrating Ability of Peptide Hormones: Key Role of Glycosaminoglycans Clustering. Int. J. Mol. Sci. 16, 27391–400 (2015). 26580613 10.3390/ijms161126025 PMC4661883

[b37] Aplin J. Embryo implantation: the molecular mechanism remains elusive. Reprod. Biomed. Online 13, 833–839 (2006). 17169205 10.1016/s1472-6483(10)61032-2

[b38] Straszewski-Chavez S. L. . The isolation and characterization of a novel telomerase immortalized first trimester trophoblast cell line, Swan 71. Placenta 30, 939–948 (2009). 19766308 10.1016/j.placenta.2009.08.007 PMC2784169

[b39] Hauk V. . Monocytes from Sjögren’s syndrome patients display increased vasoactive intestinal peptide receptor 2 expression and impaired apoptotic cell phagocytosis. Clin. Exp. Immunol. 177, 662–70 (2014). 24827637 10.1111/cei.12378 PMC4137850

[b40] Fuxman Bass J. I. . Characterization of bacterial DNA binding to human neutrophil surface. Lab. Invest. 88, 926–37 (2008). 18626469 10.1038/labinvest.2008.59

[b41] Gabelloni M. L., Trevani A. S., Sabatté J. & Geffner J. Mechanisms regulating neutrophil survival and cell death. Semin. Immunopathol. 35, 423–37 (2013). 23370701 10.1007/s00281-013-0364-x

