# Supplementary material for: Vasoactive Intestinal Peptide modulates trophoblast-derived cell line function and interaction with phagocytic cells through autocrine pathways
Source: Sci Rep. 2016 May 23;6:26364. doi: 10.1038/srep26364 (PMC4876379; doi:10.1038/srep26364)
Supplement: Supplementary Information [file srep26364-s1.pdf]

Vasoactive Intestinal Peptide modulates trophoblast-derived cell line function and interaction with phagocytic cells through autocrine pathways

Daiana Vota[1], Daniel Paparini[1], Vanesa Hauk[1], Ayelén Toro[2], Fatima Merech[1], Cecilia Varone[2], Rosanna Ramhorst[1], Claudia Pérez Leirós[1]*

Supplementary information

VPAC1 y VPAC2 expresión on trophoblast-derived cell lines

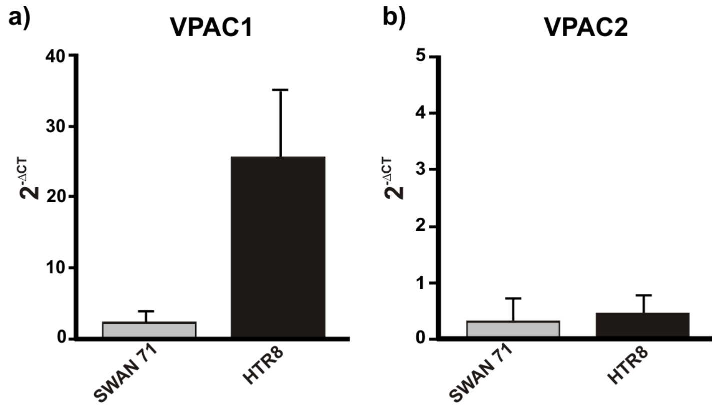

Supplementary material. Fig. S1 cDNA from SWAN 71 and HTR8 cell lines were subjected to qRT-PCR to confirm VPAC1 (a) and VPAC2 (b) expression as described in [22]; (n=3).

Plasmid transfection and VPAC2 over-expression controls

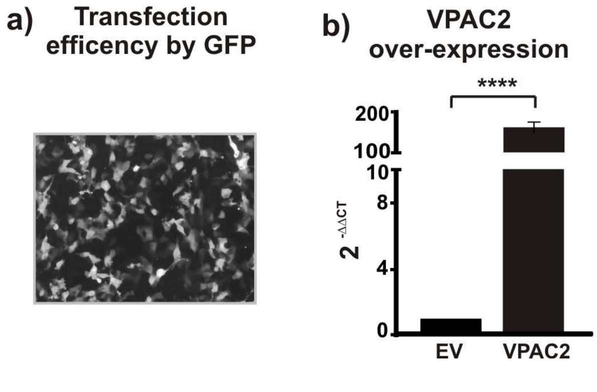

Supplementary material. Fig. S2 a) Cells were co-transfected with a VPAC2 and a GFP plasmid to evaluate the transfection efficiency. 36 h post-transfection images were taken and analyzed

using the image J program. A representative image is shown. b) VPAC2 over-expression was confirmed by qRT-PCR (****$P<0.0001$; n=4).
